# Water Availability and Air Humidity Strongly Affect Photosynthesis in Biofilms on Tree Trunks: Predictions for a Climate‐Changing World

**DOI:** 10.1111/1462-2920.70393

**Published:** 2026-07-25

**Authors:** Ana Beatriz de Lima Freitas, Tiago Vilas‐Boas, Cleber Cunha Figueredo

**Affiliations:** ^1^ Departamento de Botânica Universidade Federal de Minas Gerais Belo Horizonte Minas Gerais Brazil; ^2^ Jardim Botânico da Fundação de Parques Municipais e Zoobotânica de Belo Horizonte Belo Horizonte Minas Gerais Brazil

**Keywords:** chlorophyll fluorescence, cyanobacteria, dehydration, relative water content, tree bark

## Abstract

Climate change intensifies drought in several regions. Here, we assess how water availability and air humidity regulate photosynthesis in epiphytic cyanobacterial biofilms on *Jacaranda mimosifolia* . We combined field measurements with controlled laboratory experiments, exposing biofilms to hydration–dehydration cycles and assessing, by chlorophyll fluorescence: potential quantum yield (Fv/Fm), relative electron transport rate (rETR_max_), and photosynthetic photon flux density at saturation (PPFD_sat_). Historical precipitation and relative humidity (RH) data from the last six decades were analysed. Biofilms were completely inactive under dry conditions (Fv/Fm = 0), but rapidly recovered photosynthesis (Fv/Fm**≈**0.5) within two hours of rehydration. A bark water content of approximately 16% was sufficient to restore 50% of Fv/Fm, whereas higher water availability (> 30%) was required to recover rETRmax and PPFDsat. Air humidity influences the duration of water availability, whereas photosynthetic reactivation depends primarily on liquid water. Climate analyses (1961–2024) revealed an increase in the local frequency of drier days (RH < 60%; Vapour Pressure Deficit > 1; rainfall = 0 mm). This trend suggests that these biofilms are increasingly exposed to prolonged water stress, potentially compromising their ecological functioning and associated ecosystem services. Overall, our findings highlight the vulnerability of epiphytic cyanobacterial biofilms to ongoing climatic drying.

## Graphical abstract

Photosynthetic behaviour in cyanobacteria‐dominated biofilms on tree bark is very sensitive to water availability and vulnerable to climatic changes.

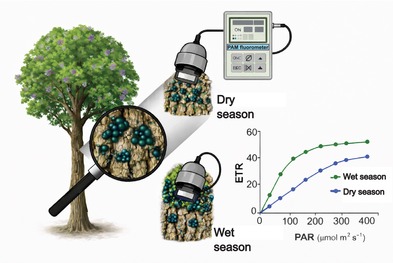

## 1 Introduction

Climate change has relevantly impacted ecosystems through shifts in temperature (Pandit and Sharma 2023) and hydrological regimes, changing rainfall and atmospheric moisture patterns (Konapala et al. 2020; Jones and Ricketts 2022). These alterations have led to an increase in extreme events, such as floods and prolonged droughts (Dai 2011; Trenberth 2011), affecting multiple terrestrial environments and disrupting ecological processes essential for sustaining life (Allison 2023). In tropical and subtropical regions dry seasons have become longer and are marked by more intense droughts (van Loon 2015; Hao et al. 2018). Close to latitude 20° S, the Brazilian southeast region experienced historic droughts between 2013 and 2015, with important environmental and socioeconomic consequences (Coelho et al. 2016; Dias et al. 2018). Since plants are considered especially sensitive to droughts (Jones and Ricketts 2022), most assessments of abiotic stress impacts have been focused on these organisms, particularly on angiosperms due to the high interest in crops (Hoekstra et al. 2001; Farrant and Moore 2011). However, there is a lack of studies examining the responses of other photoautotrophic groups such as bryophytes, and terrestrial algae or cyanobacteria (Oliver et al. 2005; Holzinger and Karsten 2013; Billi and Potts 2002; Peñaloza‐Bojacá et al. 2023).

Since microbial communities have a key role in ecosystem functioning, contributing to the maintenance of other life forms (Cavicchioli et al. 2019; Oliveira et al. 2024), it is essential to understanding how climate change would impact these organisms. Microbial communities in terrestrial ecosystems and, consequently, their ecological functions are sensitive to abiotic stresses, particularly to sudden changes in temperature and moisture (Lüttge and Büdel 2010; Allison 2023; Fu et al. 2023; Xu et al. 2023). These microorganisms are not only affected by climate but are also key regulators of climate processes due to their fundamental role in carbon dynamics (Cavicchioli et al. 2019). In addition to this role, they are also relevant in nutrient cycles, with cyanobacteria playing a fundamental role in the early stages of ecological succession in terrestrial ecosystems (Maltsev et al. 2022). They enrich the system with nitrogen (Abrantes et al. 2023) and organic matter, improving the process of colonisation by other organisms (Schmidt et al. 2008). Extensive research has been conducted on the impacts of climate change on cyanobacteria on aquatic environments (Gobler 2020; Hallegraeff 2010; Paerl et al. 2016), leaving a gap regarding their responses in terrestrial ecosystems.

The spores of terrestrial cyanobacteria can be highly resistant to desiccation, but the vegetative forms exhibit varying levels of sensitivity to water availability (Raanan et al. 2016; Ferreira et al. 2025). Cyanobacteria associated with terrestrial substrates are commonly poikilohydric organisms, whose metabolic activity strongly depends on water availability. Unlike vascular plants, these organisms do not actively regulate their internal water status and may tolerate complete desiccation by entering a metabolically inactive state (Oliver et al. 2005). However, the few studies that focused on photosynthesis in cyanobacterial lichens and isolated cyanobacteria demonstrated that the recovery of photosynthetic activity after desiccation generally requires direct contact with liquid water, whereas high atmospheric humidity alone is often insufficient to reactivate photosynthesis (Lange et al. 1986, 1989; Leisner et al. 1996). This physiological feature differs from patterns reported for many poikilohydric green algae and has important implications for understanding the responses of terrestrial cyanobacterial biofilms to drought conditions.

The major impacts of water scarcity on cyanobacteria are genome fragmentation, weakening of the plasma membrane, discoloration of phycobiliproteins, accumulation of reactive oxygen species (ROS), loss of respiratory activity after rehydration (Billi 2009), and general alterations in photosynthetic metabolism (Hirai et al. 2004; Rajeev et al. 2013). However, these microorganisms occur in a variety of terrestrial substrates subject to desiccation, including tree barks, where they form biofilms (Lemes‐da‐Silva et al. 2012). The ecological roles of cyanobacteria dominated biofilms growing on tree trunks are still poorly understood. Some studies have reported significant changes in the chemical composition of rainwater leaching tree surfaces, including its enrichment in nutrients, such as nitrogen and carbon (Schroth et al. 2001; Chuyong et al. 2004; Neustupa and Škaloud 2008; Ponette‐González 2021; Jiang et al. 2021; Lima et al. 2024). However, these investigations generally did not address the potential contribution of microorganisms inhabiting the bark to this enrichment process. As observed for rock surfaces (Abrantes et al. 2023), further studies on the role of cyanobacteria biofilms associated with tree trunks on this nutrient enrichment are essential, and it is also necessary to understand how abiotic factors modulate physiological aspects of the microbial communities growing on tree bark.

Although previous studies have investigated the effects of desiccation on cyanobacteria from aquatic systems and on poikilohydric organisms such as lichens and terrestrial green algae (Potts 1994, 1999; Holzinger and Karsten 2013; Gasulla et al. 2021), little is known about the physiological responses of corticolous cyanobacterial biofilms to water limitation. In particular, there is a lack of information regarding how variations in bark water content and atmospheric humidity affect the photosynthetic performance of cyanobacteria‐dominated biofilms growing on tree bark under drought conditions. Understanding these responses is essential because these microbial communities may contribute to nutrient dynamics and ecosystem functioning in tropical forests, as observed for tropical soils (Abrantes et al. 2023), and would be impacted by prolonged dry periods under climate change scenarios. The present study aimed to assess how distinct levels of water availability influence the photosynthetic activity of cyanobacteria dominated biofilms on tree bark. Our hypothesis is that the photosynthetic activity of cyanobacteria‐dominated biofilms is directly dependent on bark hydration status and water availability, with atmospheric humidity influencing dehydration dynamics and water retention on the bark surface. We also evaluated the climate data over the last decades to assess the water availability and relative air humidity tendency in the region through the years, and if the increase in drier days frequency could represent a threat to these biofilms. The results may provide insights into the conservation of terrestrial ecosystems impacted by climate change conditions, such as extended droughts predicted, and support the development of mitigation strategies in vulnerable regions.

## 2 Material and Methods

### 2.1 Study Area, Plant and Biofilm Material

The study was carried out with epiphytic biofilms covering the bark of *Jacaranda mimosifolia* D. Don (Bignoniaceae) trees, located on the campus of the Federal University of Minas Gerais (UFMG), at Belo Horizonte city, Brazil (19°52′ S; 43°57′ W). This species was selected due to its high abundance in the urban area studied, the very common presence of cyanobacterial biofilm on its thick bark, which detaches easily, facilitating sampling. The area is characterised by a subtropical highland climate (Cwb), with a warm and humid season (October–March) and a dry and cold season (April–September) (Alvares et al. 2013). Samplings were conducted at the end of the rainy season, with in situ measurements occurring between March 26 and April 1. The barks of *J. mimosifolia* trees were essentially covered by a dark‐layered biofilm, suggesting cyanobacterial prevalence, while layers containing lichens or bryophytes were excluded from all evaluations to minimise measurement uncertainty.

Seven plants were selected, and three bark fragments (~5 cm^2^) from each plant were sampled in the morning (8:00–10:00 AM), placed in plastic bags, and immediately taken to the laboratory. Samples from the same tree were collected at least 30 cm apart to increase the representativeness of local variability and to avoid sampling highly similar communities derived from a continuous biofilm layer. Each bark fragment was divided into two subsamples, with one of them being used for community composition evaluation. For this purpose, a suspension was prepared from each of these subsamples by removing the biofilm via gentle brushing in distilled water, resulting in 21 suspension samples. The material was examined under an optical microscope (Olympus CH‐30) for organism identification. Given the limitations of morphology‐based taxonomy in algae and cyanobacteria, identification was restricted to the genus level, and potential variation in morphotypes within genera was taken into account.

### 2.2 Photosynthetic Activity Assessment

To evaluate whether biofilms were metabolically active and the level of this activity, photosynthesis was quantified both in situ and in vitro through chlorophyll fluorescence measurements using a MINI‐PAM fluorometer (Walz, Effeltrich, Germany). For this purpose, the other subsample of each bark sample was dark‐adapted for 20 min in black covered polypropylene containers (11 × 11 × 3.5 cm) in situ and in laboratory conditions (~25°C, RH~60%), and the maximum quantum yield of photosystem II (Fv/Fm) was determined (Bjorkman and Demmig 1987). Rapid light curves (RLC) were also performed by increasing actinic light in nine steps at 20s‐intervals, from 0 to 1500 μmol m^−2^ s^−1^. Samples in situ were evaluated under natural weather conditions and covered from direct sunlight, avoiding light intensity variation, while in vitro analyses were performed under laboratory‐controlled conditions. From these curves, the maximum relative electron transport rate (rETRmax), the saturating photosynthetically active radiation (PPFDsat) and the effective quantum yield at the PPFDsat (ΔF/Fm'sat) were calculated according to Rascher et al. (2000). The use of relative electron transport rate (rETR) instead of absolute ETR values was chosen in this study because our primary objective was to evaluate light response curve trends, and measuring the actual absorbance of these biofilms is technically unfeasible due to the morphophysiological particularities, which are not clearly understood or mentioned in the literature. Relative electron transport rate (rETR) data were fitted to the exponential saturation model rETR = a(1 − e^−bPPFD^), where *a* represents the asymptotic maximum rETR and *b* describes the initial slope of the light‐response curve, following the approach described by Ralph and Gademann (2005). Saturating irradiance (PPFDsat) was defined as the irradiance at which rETR reached 90% of rETRmax. The effective quantum yield of PSII at saturating irradiance (ΔF/Fm'sat) was estimated by interpolation of the observed ΔF/Fm’ values at PPFDsat. RLC were analysed separately for each individual replicate. Nonlinear regression models were fitted to rETR versus PPFD data using the Levenberg–Marquardt optimisation algorithm implemented in the nlsLM function of the *minpack.lm* package. Curve fitting was automated across replicates using functions from the *dplyr* package. All analyses were conducted in R (version 4.2.4).

### 2.3 In Situ Evaluation of Photosynthesis and Water Relations in the Biofilms

To evaluate the photosynthetic activity in the biofilm and its behaviour under natural environment variation, mainly focused on precipitation, relative humidity (RH), and vapour pressure deficit (VPD), in situ analyses were performed on March 26th, 27th, 29th, and April 1st, corresponding to a transitional period from rainy to dry season, with high variation in humidity and precipitation values. Maximum quantum yield (Fv/Fm) and RLC were evaluated as described previously, with the measurements being performed in situ in two bark fragments immediately sampled from five different trees (*n* = 10 samples per day). rETRmax, PPFDsat, and ΔF/Fm'sat were also determined. After that, samples were placed in plastic bags and taken to the laboratory to determine their relative water content (RWC). For this, fresh weight (FW) was immediately recorded in the laboratory and then samples were submerged in distilled water, stored at 4°C in the dark and weighed after 96 h to determine their turgid weight (TW). Dry weight (DW) was obtained by oven‐drying the samples at 40°C until a constant weight was achieved. RWC was then calculated using the following formula:

$$\mathrm{RWC}\left(\%\right)=\left(\frac{\left(\mathrm{FW}-\mathrm{DW}\right)}{\left(\mathrm{TW}-\mathrm{DW}\right)}\right)x\;100$$

where FW is the fresh weight; DW is the dry weight; and TW is the turgid weight.

### 2.4 In Vitro Measurements: Hydration and Dehydration Cycle

In the laboratory, the 21 barks were subjected to a hydration cycle during 5 days followed by a dehydration cycle of 10 days in a Biochemical Oxygen Demand (B.O.D.) incubator (model 710, Thoth, Brazil) set at 25°C with a 12‐h photoperiod (70–100 μmol m^−2^ s^−1^) and ~55% RH. Before starting the hydration cycle, the samples were weighed using a precision analytical balance (Shimadzu AUW220D), and then placed into transparent polypropylene containers (11 × 11 × 3.5 cm), which were kept closed but were not airtight, each containing the three replicates from a single tree (*n* = 7 trees). At the beginning of the hydration cycle, 10 mL of distilled water was directly applied onto the bark surface to ensure complete wetting of the cyanobacterial layer. During the hydration cycle (120 h), the residual water accumulated at the bottom of each container was removed and replaced daily with 10 mL of fresh distilled water, which was again directly applied onto the bark samples to prevent excessive microbial growth and sample degradation. The weight of each bark sample was measured after 2, 8, 24, 48, 72, 96, and 120 h from the beginning of the experiment. After 120 h of hydration, the transparent polypropylene containers were dried with paper towels, and the bark samples were subjected to a dehydration cycle in the B.O.D. incubator by completely interrupting the water supply for the following 10 days. During this phase, the samples were weighed every 24 h. Relative humidity (RH) inside each container was also measured daily using a portable thermo‐hygrometer (AKSO AK635, Brazil). The vapour pressure deficit (VPD) in each container was calculated based on RH and temperature values, according to the Monteith and Unsworth (2013) formula:

$$\mathrm{VPD}=(0.611\times \left(\exp\;\;\left(\frac{\left(17.27\times \text{Tair}\right)\;}{\left(237.3+\text{Tair}\right)\;}\right)\times \left(1-\left(\frac{\mathrm{RH}}{100}\right)\right)\right)$$

where Tair is the air temperature (°C) and RH is relative humidity (%).

The relative water content of bark samples, calculated based on the RWC formula, was evaluated during the 15 days of incubation. We considered the dry weight (DW) as the weight measured on the last day, after complete desiccation. Fresh weight (FW) was defined as each weight recorded immediately after the sampling and during incubation, and turgid weight (TW) was defined as the maximum weight reached by each sample along the experiment. All the photosynthetic parameters measured in situ were also assessed at the same time points as the weight measurements, except at 120 h, when the condition was changed to the desiccation period with no water addition.

### 2.5 Survey of the Historic Series of Data of Relative Air Humidity Variation in Belo Horizonte

To characterise historical climatic trends related to water availability, daily relative humidity (RH) (1961–2024) and precipitation data (1961–2023) for Belo Horizonte were obtained from a meteorological station of the National Institute of Meteorology (INMET) located approximately 7 km from the study site. Temporal trends were evaluated based on the annual number of days with different levels of total precipitation: 0, < 2, < 5, < 10 and < 20 mm. Although the photosynthesis in cyanobacteria dominated biofilms should mainly depend on liquid water availability (Lange et al. 1986; Lange et al. 1989; Leisner et al. 1996), relative humidity and VPD values were also considered, as they influence moisture retention and may show greater temporal variability. Both relative humidity and VPD were calculated as daily mean values, which were subsequently averaged to obtain annual mean values. Based on our laboratory findings, a critical RH threshold was identified above which photosynthetic activity in the biofilms is significantly affected, albeit indirectly. This threshold was then used to classify the historical record into wetter and drier periods, allowing the identification of the major regional trends.

### 2.6 Statistical Analysis

Data obtained in situ (Fv/Fm, ΔF/Fm'sat, rETRmax, PPFDsat, and RWC) were analysed considering five replicates, and the predictor variable was sampling day (March 26, 27, 29, and April 1). Normality and homoscedasticity were tested using the Shapiro–Wilk and Bartlett tests (*p* < 0.05), respectively. A one‐way ANOVA was performed for the in situ data, as the same fragments were evaluated over time, followed by Tukey's test (*p* < 0.05) when appropriate. Data that did not meet assumptions of normality and homogeneity of variances were analysed using the non‐parametric Kruskal–Wallis test, followed by Dunn's post hoc test with multiple comparisons (*p* < 0.05). For the in vitro data, a repeated‐measures one‐way ANOVA was applied, since the same samples were evaluated, and Tukey's test (*p* < 0.05) was also used for multiple comparisons. These statistical analyses and figure preparation were performed in GraphPad Prism 8 (San Diego, CA, USA). Relationships between photosynthetic variables (Fv/Fm, ΔF/Fm'sat, rETRmax, and PPFDsat) and water‐related variables (RH, VPD and RWC) were assessed by nonlinear regressions for both in vitro and in situ analyses. However, the relationship between RH and photosynthetic variables was evaluated only for the in vitro experiment, as a single RH value per day was obtained for all samples in the in situ analysis. We fitted sigmoidal models and from these models, we obtained the W50 value, defined as the percentage of RWC, RH and VPD of the bark–biofilm complex required to activate 50% of the photosynthetic apparatus according to the four photosynthetic variables. The significance of sigmoidal regressions was tested using the *F*‐test. The overall temporal trend of climate data (precipitation, RH, and VPD) between 1961 and 2024 was assessed using linear regression analyses. These analyses were carried out using the ‘*stats*’ and ‘*drc*’ packages in R, version 4.2.2 (R Core Team 2022). Figures were generated with GraphPad Prism 8 (San Diego, CA, USA).

## 3 Results

### 3.1 Characterisation of the Cyanobacterial Community on Bark Samples of *Jacaranda mimosifolia*

**FIGURE 1:**
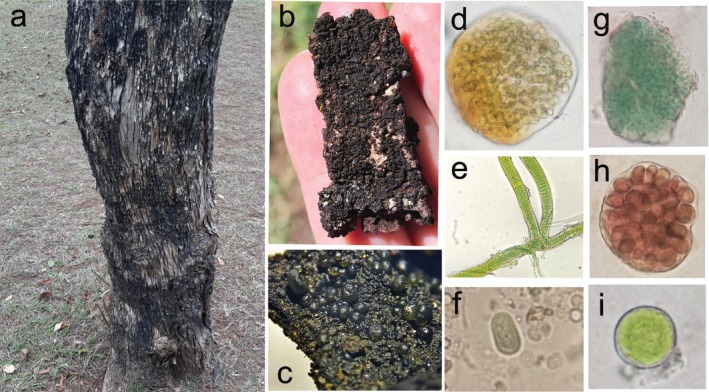
*Jacaranda mimosifolia* tree (a), sampled bark fragments (b), *Nostoc* colonies on bark visualised under a stereomicroscope (c), and genera of photosynthetic microorganisms identified by optical microscopy in the biofilm samples: *Nostoc* (d), *Scytonema* (e), *Cyanobium* (f), *Chlorogloeopsis* (g), *Asterocapsa* (h), and *Trebouxia*, a green alga (Chlorophyta) often associated with lichens (i).

All the bark samples were covered by biofilms (Figure 1a–c) with a similar composition, with predominance of the cyanobacteria genera *Nostoc* (Figure 1d), *Scytonema* (Figure 1e), *Cyanobium* (Figure 1f), and *Chlorogloeopsis* (Figure 1g), and Asterocapsa (Figure 1h). Few samples showed a very rare occurrence of the chlorophyte *Trebouxia* (Figure 1i). Other detected non‐abundant microorganisms included filamentous fungi.

### 3.2 In Situ Dynamics of Photosynthetic Activity in Biofilms Under Different Moisture Conditions

**FIGURE 2:**
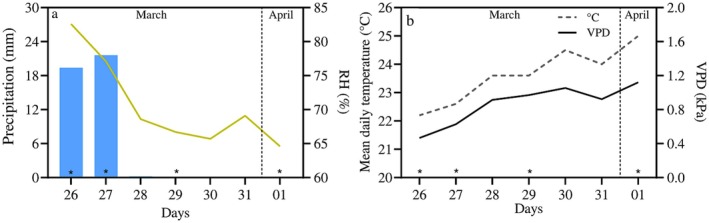
Climatic conditions in the study area during the study period, based on data obtained from the National Institute of Meteorology (INMET). (a) Daily precipitation (blue bars) and relative humidity (yellow line). (b) Mean daily temperature (dotted line) and mean daily vapour pressure deficit (VPD; solid line) on March 26, 27, and 29, and April 1, 2025 (*).

**FIGURE 3:**
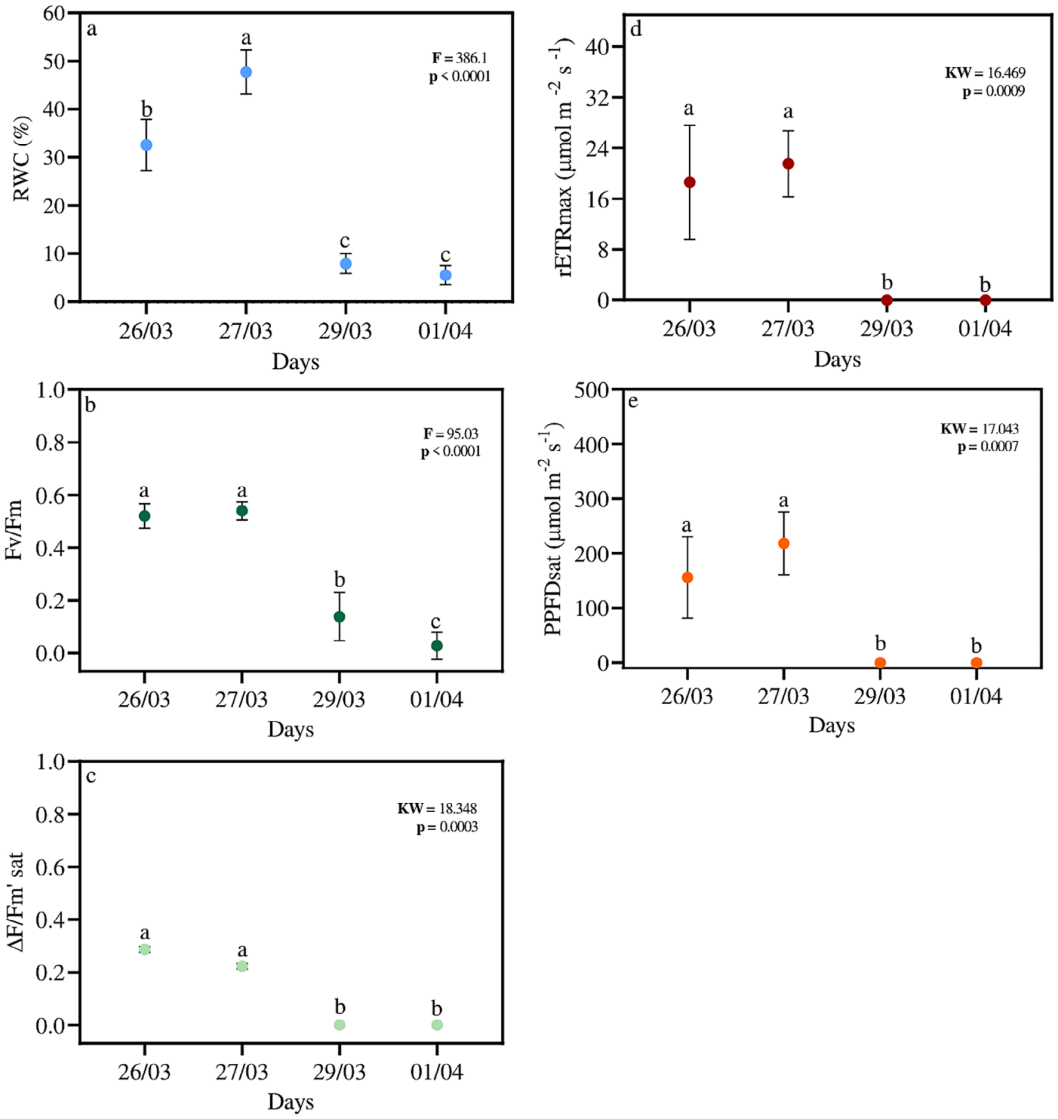
Relative water content (RWC—blue dots, a), potential quantum yield of photosystem II (Fv/Fm—dark green dots, b), effective quantum yield of PSII at saturating irradiance (ΔF/Fm'sat—light green dots, c), relative maximum electron transport rate (rETRmax—red dots, d), and saturating photosynthetically active radiation (PPFDsat—orange dots, e), of bark‐biofilm complex from *Jacaranda mimosifolia* collected on March 26, 27, and 29, and April 1, 2025. Different letters indicate statistically significant differences according to one‐way ANOVA (F) followed by Tukey test (*p* < 0.05), or Kruskal‐Wallis (KS) followed by Dunn's test (*p* < 0.05); KS = (p < 0.05). Symbols represent mean ± standard deviation.

The climate conditions were very different among the sampling days, with March 26 and 27 being humid (ca. 20 mm precipitation and relative humidity higher than 75%) compared to March 29 and April 1, which showed no precipitation and RH close to 65% (Figure 2a). Daily mean temperature on the rainy days 26 and 27 was between 22.2°C and 22.6°C, respectively, while on day 29 it was 23.6°C, and 25.0°C on April 1 (Figure 2). VPD followed the same pattern as temperature in the sampling days, increasing from 0.46 and 0.63 (on March 26 and 27) to 0.97 and 1.12 (on March 29 and April 1) (Figure 2b). The relative water content (RWC) of barks varied significantly among these dates, with values reaching 33% and 47% on March 26 and 27, respectively, while RWC was lower than 10% on March 29 and April 1 (Figure 3a). On March 26 and 27, Fv/Fm values were 0.52 and 0.57, respectively, while the values declined to approximately 0.10 and 0.05 on March 29 and April 1 (Figure 3b). ΔF/Fm'sat average was 0.29 and 0.22 on March 26 and 27, respectively, and decreased to zero on March 29 and April 1 (Figure 3c). The rETRmax average values of 18.6 and 21.5 μmol m^−2^ s^−1^ were recorded on the wetter days, March 26 and 27, respectively, but decreased to zero on March 29 and April 1 (Figure 3d). The same trend was observed for PPFDsat, which was 156 and 218 μmol m^−2^ s^−1^ on March 26 and 27, respectively, and decreased to zero on March 29 and April 1 (Figure 3e).

### 3.3 Dynamics of Photosynthetic Recovery in Biofilms Under Different Moisture Conditions

**FIGURE 4:**
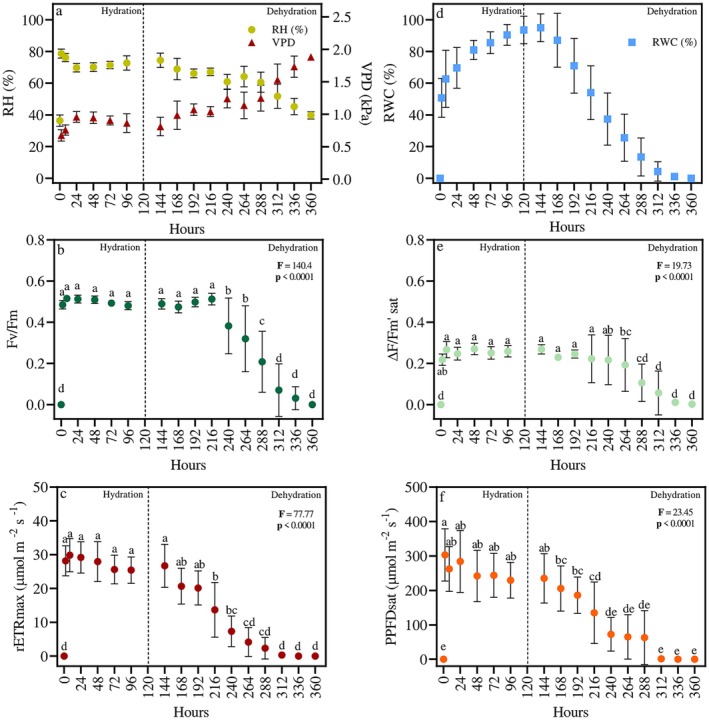
Relative humidity (RH) and vapour pressure deficit (VPD) (a) at the sample containers and relative water content (RWC) of bark samples throughout the in vitro experiment (*n* = 7) (d). Potential quantum yield of photosystem II (Fv/Fm—dark green dots, b), effective quantum yield of PSII at saturating irradiance (ΔF/Fm'sat—light green dots, e), relative maximum electron transport rate (rETRmax—red dots, c), and saturating photosynthetically active radiation (PPFDsat—orange dots, f) in bark–biofilm complex over time (hours) during a hydration–dehydration cycle at 25°C under a 12/12 h light/dark photoperiod. Dotted vertical line represents interruption of water replenishment after 120 h to initiate the desiccation period. Symbols represent mean ± standard deviation. Different lowercase letters indicate statistically significant differences among means according to one‐way ANOVA followed by Tukey's test (*p* < 0.05).

A rapid increase in sample weight was recorded during the initial hours of hydration, peaking at approximately 120 h, when the dehydration cycle started, and after 168 h a gradual decline in RWC was detected, reaching minimum values at the end (360 h) of the experiment (Figure 4d). Relative air humidity inside the containers ranged from 30% to 80%, with a slow decrease occurring only 4 days after the interruption of hydration and a opposite trend in VPD (Figure 4a).

The photosynthetic activity was zero (Fv/Fm = 0) immediately after sampling, in the desiccated state, but it significantly recovered after only 2 h of hydration, with Fv/Fm reaching approximately 0.5 (Figure 4b). Between 2 and 216 h, Fv/Fm remained stable, fluctuating between 0.45 and 0.55. A significant and gradual decline began after 240 h, returning to near‐zero levels by 312 h and reaching, at 360 h, a state similar to that observed prior to hydration (Figure 4b). For ΔF/Fm'sat, the same pattern occurred as Fv/Fm; ΔF/Fm'sat of 0.22 was obtained after 2 h and 0.27 after 8 h of hydration, with average values similar to or slightly higher being observed until 240 h, when a significant decrease started (Figure 4e). Even though relative humidity affects the maintenance of liquid water availability in the system, while not directly affecting the photosynthesis of cyanobacteria in biofilms, the onset of a significant drop in photosynthetic activity coincided with RH values < 60%. Thus, this percentage was adopted as critical for evaluating temporal variations in RH in the region over the last few decades and may be an additional factor in determining the lack of liquid water in the system.

The maximum relative electron transport rate (rETRmax) increased rapidly with hydration, reaching on average 28 μmol m^−2^ s^−1^ after only 2 h of hydration and maintaining rETRmax average above 26 μmol m^−2^ s^−1^ until 144 h, when a gradual reduction started and levels close to zero were detected after 288 h (Figure 4c). The same trend was observed for PPFDsat, which increased to 300 μmol m^−2^ s^−1^ within the first 2 h, maintaining values above 200 μmol m^−2^ s^−1^ until 144 h, and subsequently decreasing to zero by the end of the experiment (Figure 4f).

**FIGURE 5:**
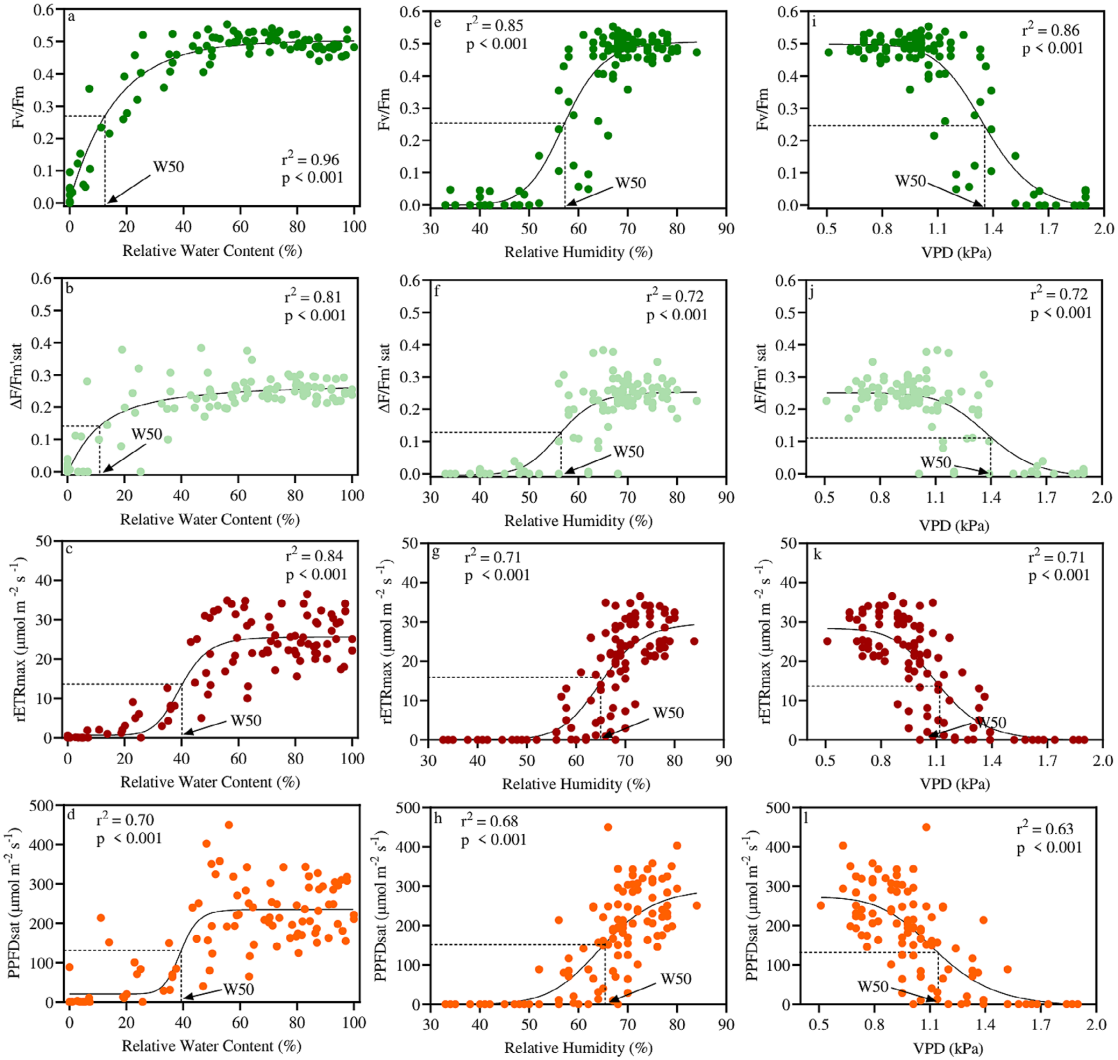
Relationship between the relative water content (RWC, left panels a–d), relative humidity (RH, middle panels e–h) and vapour pressure deficit (VPD, right panels i–l) and potential quantum yield of photosystem II (Fv/Fm—dark green dots, a, e, i), effective quantum yield of PSII at saturating irradiance (ΔF/Fm'sat—light green dots, b, f, j), relative maximum electron transport rate (rETRmax—red dots, c, g, k), and saturating photosynthetically active radiation (PPFDsat—orange dots, d, h, l) at 25°C under a 12/12 h light/dark photoperiod. The curves represent the nonlinear fits used to estimate W50 values, that is, the point at which each parameter reaches 50% of its maximum response.

The W50 for Fv/Fm and ΔF/Fm'sat was reached at only 12% and 11% of bark‐biofilm RWC, respectively (Figure 5a,b), whereas 40% RWC was required to reach the W50 of the rETRmax and PPFDsat parameters (Figure 5c,d). The RH required for Fv/Fm and ΔF/Fm'sat to reach their W50 was 57% and 56%, respectively (Figure 5e,f), while both rETRmax and PPFDsat reached their W50 values at approximately 65% RH (Figure 5g,h). For VPD, W50 for Fv/Fm and ΔF/Fm'sat was reached at 1.35 kPa and 1.39 kPa, while for rETRmax and PPFDsat it was around 1.11 kPa and 1.14 kPa (Figure 5i–l).

**FIGURE 6:**
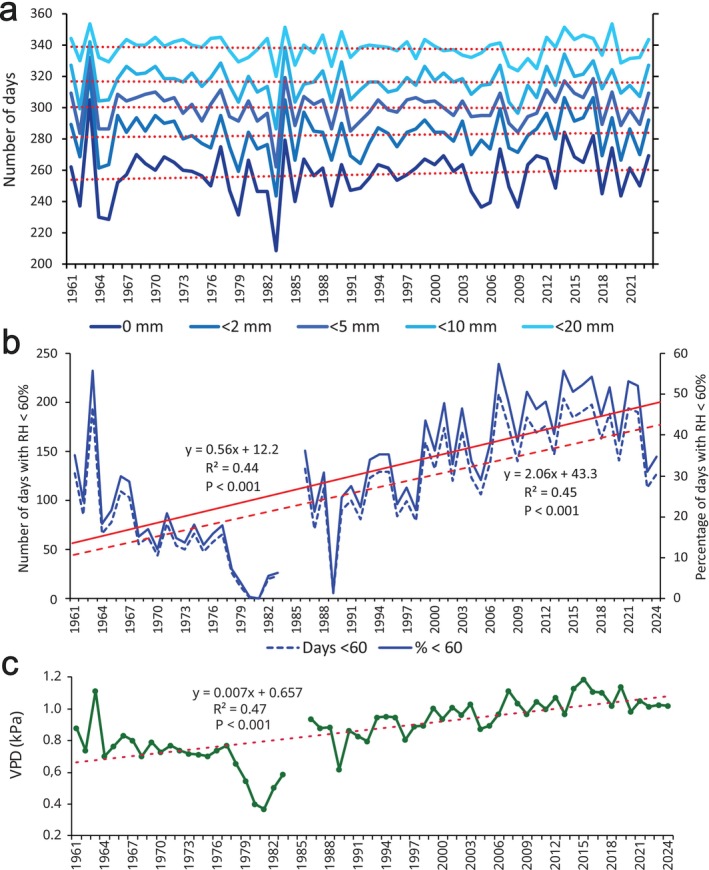
Historical climatic trends related to water availability in Belo Horizonte. Number of days by year (1961–2023) with total precipitation at different levels (0–20 mm; temporal trend in red dashed lines, but the regression models were not statistically significant and are not suitable for reliable predictions) (a); Annual absolute number (dashed line) and percentage (solid line) of days with a daily mean relative humidity below 60% between 1961 and 2024 (b); Annual mean vapour pressure deficit (VPD; a measure of atmospheric water demand), calculated as the average of the daily mean values for each year, from 1961 to 2023. The data indicate an increasing frequency of drier days and VPD values over the last decades.

Although the number of days per year with lower precipitation (0 and < 2 mm) showed an increasing trend from 1961 to 2023 (Figure 6a), the linear regression showed low explanatory power (*R* ^2^ < 0.1) and no statistical significance (*p* > 0.05). There was considerable variation in the number of days with relative humidity (RH) below 60% in Belo Horizonte between 1961 and 2024 (Figure 6b). The early decades of the record, particularly from the 1960s to the 1980s, exhibited marked fluctuations. During this period, only 1 year (1963) recorded more than 200 days with RH below 60%, and a decreasing trend in the occurrence of these drier days was observed, reaching zero days in both 1980 and 1981. From the 1990s onward, however, a progressive increase in both the absolute number and the proportion of drier days per year was detected. This increase became particularly evident in the 2000s, when the number of days with RH < 60% stabilised at a high level (around 200 days per year) with reduced interannual variation. The trends of lower water availability or water stress were reinforced by VPD data, which showed a marked increase in recent decades, frequently exceeding 1.0 kPa, whereas values in the 1970s and 1980s were typically below 0.8 kPa (Figure 6c).

## 4 Discussion

Compared to soil biocrusts, where cyanobacteria have a relevant role as pioneer organisms contributing to ecological succession (Belnap et al. 2001), corticolous cyanobacteria inhabit more heterogeneous and desiccation‐prone substrates, requiring specialised adaptations (Büdel et al. 2016) and representing an underexplored source of tropical biodiversity and ecological novelty (Lemes‐da‐Silva et al. 2012). Research on photosynthetic microorganisms in terrestrial biofilms in general remains scarce in tropical regions (Dal‐Ferro et al. 2024) and is even more limited for communities associated with tree bark. Thus, the identification of the main genera in this study represents a novel contribution for future research in low latitudes. Lüttge and Büdel (2010) evaluated photosynthetic responses of a community of autotrophic microorganisms on tree bark in Germany, and they always detected a predominance of Chlorophyta species. Our results showing the prevalence of cyanobacteria over other autotrophic microorganisms in epiphytic tree biofilms are consistent with the results of Fürnkranz et al. (2008), although they focused only on leaves (epiphillic biofilms). Since cyanobacteria can release nitrogen into the surrounding environment (Abrantes et al. 2023), the predominance of these microorganisms on the trunk surface should be better understood to evaluate whether they contribute to plant nutrition. Some studies on subaerophytic cyanobacteria mention that this phylum is remarkably resistant to environmental stressors and water availability fluctuation (Potts 1999; Dreyling et al. 2022; Ferreira et al. 2025). Physiological and biochemical mechanisms, such as the production of thick mucilage (Potts 1994) by genera such as *Nostoc*, *Cyanobium* and *Asterocapsa*, photoprotective compounds (e.g., scytonemin) and efficient DNA repair systems, are evolutionary strategies that enable the survival of cyanobacteria on exposed surfaces like rocks, soil, and tree bark (Sinha and Häder 2008). Particularly for *Nostoc*, a genus commonly found in our samples, some studies highlight its high tolerance to desiccation and UV radiation (Hoffmann 1989; Sinha and Häder 2008). *Nostoc* and *Scytonema*, both commonly found in our samples, may present copious mucilage and cell differentiation (akinetes and heterocysts), which provide protection against dehydration and radiation while also enabling atmospheric nitrogen fixation (Potts 1999; Mazur and Ślesak 2025; Shang et al. 2025). The only eukaryotic microorganism in the samples (*Trebouxia*) is a chlorophyte whose resistance to stressful environments is commonly associated with its symbiotic relationship with fungi in lichens (Garty 2001), although, when isolated, it can tolerate desiccation and recover Fv/Fm to values close to initial levels after rewetting (Wieners et al. 2012). In summary, considering that the biofilms live under stressful conditions and that they can be functionally relevant for the host plants, it is necessary to better understand their resistance to climate change, especially under a scenario of increasing drought frequency.

For the in situ evaluations, sampling during a transitional period between the dry and rainy seasons proved to be adequate to evaluate the effects of water on biofilm photosynthetic activity, since large fluctuations in water availability were detected. These fluctuations caused RWC to vary by a factor of three between the driest and rainiest days, showing large variations in water retention within the bark–biofilm complex associated with *J. mimosifolia* trunks. Rainfall events were associated with a substantial increase in Fv/Fm, ΔF/Fm'sat, rETRmax, and PPFDsat, indicating rapid physiological reactivation after hydration. Photosystem II (PSII) activity is widely recognised as a sensitive indicator of the functional integrity of the photosynthetic apparatus (Gomes et al. 2021), and the fluorescence parameters indicated that metabolism reactivation occurred very rapidly after biofilm rehydration. In fact, Fv/Fm returned to typical levels for cyanobacteria (0.5–0.6) (Lan et al. 2012, 2019) after only 2 h, which suggests a highly efficient desiccation tolerance mechanism in the photosynthetic apparatus, as also observed by Lüttge et al. (1995) and Harel et al. (2004) for terrestrial cyanobacteria.

Since one of our goals was to evaluate the effects of water availability on photosynthesis and to connect it with climate conditions, the data obtained in vitro allowed us to attempt to determine the limiting values of RWC, VPD, and RH for photosynthetic activity in tree bark biofilms. The similar results for W50 obtained in the laboratory tests reinforced the conclusion that Fv/Fm and ΔF/Fm'sat were the most tolerant parameters to dehydration, while rETRmax and PPFDsat required higher hydration levels (40% RWC) for 50% recovery of photosynthetic activity. This pattern is consistent with previous studies showing that Fv/Fm is generally more tolerant to dehydration and recovers earlier than electron transport–related parameters in cyanobacteria (Strong et al. 2013; Raanan et al. 2016; Yadav et al. 2023). These results show that while the quantum efficiency of PSII (Fv/Fm) responds rapidly to initial hydration, electron transport and light‐use capacity are only effectively restored under more favourable moisture conditions (Harel et al. 2004; Raanan et al. 2016).

The fact that bark dehydration occurred much more gradually than the hydration resulted in a slower decline of both rETRmax and Fv/Fm, beginning only 72 and 120 h after the water supply was interrupted, respectively, and returning to zero levels only after 10 days. This behaviour suggests an ability to immediately use the available water from the first rains and provides a more adequate time window to allow the associated microorganisms to be metabolically prepared when the drier period starts. Some studies show that the water content in tree barks can rise due to both high RH and water supplied by rainfall, with the rate of water absorption increasing across humidity levels regardless of the species evaluated (Tonello et al. 2025). Although RH, VPD, and RWC were analysed separately in this study, these variables are physically interconnected because atmospheric conditions directly regulate water exchange between bark surfaces and the surrounding air. Relative humidity influences the potential for water retention and vapour‐phase hydration, whereas VPD integrates the combined effects of temperature and humidity on atmospheric evaporative demand (Monteith and Unsworth 2013). Consequently, high RH and low VPD reduce water loss from bark surfaces and may allow limited hydration of both bark tissues and the associated biofilm matrix (Potts 1994; Davila et al. 2013). In contrast, increasing VPD enhances evaporation and accelerates bark desiccation, reducing the period during which photosynthetic organisms remain metabolically active. This pattern was evident in our field observations, where the decline in photosynthetic activity coincided with an increase in VPD from approximately 0.5–0.6 kPa under rainy conditions to values close to 1.0–1.1 kPa during dry periods. Likewise, the W50 values obtained for photosynthetic parameters ranged from approximately 1.1–1.4 kPa, indicating that relatively small increases in atmospheric evaporative demand can substantially reduce photosynthetic performance in bark‐associated cyanobacterial communities. Hydration through atmospheric water vapour alone is ineffective and generally slower than direct liquid water inputs provided by rainfall, dew, or stemflow, which explains why RWC remained the strongest predictor of photosynthetic recovery despite the significant relationships observed with RH and VPD (Lange et al. 1986; Harel et al. 2004). Together, these results are the first evidence that photosynthetic activity in corticolous biofilms is controlled not only by the amount of water stored in the bark but also by atmospheric conditions that regulate water retention and loss, emphasizing the importance of considering both substrate hydration and evaporative demand when evaluating the ecological functioning of these communities. Thus, it is important to consider the connections among climate conditions affecting water availability in tree bark and their effects on the activity of the biota associated with tree trunks. This is particularly relevant because microorganisms, including cyanobacteria, may act as sources of organic matter and nitrogen for the surrounding environment, contributing to host plant nutrition (Rai et al. 2000). Considering climate change projections, it is necessary to begin understanding how these environmental modifications will impact these communities, their interactions, and especially their ecological functions.

Given the high variability and stochastic nature of precipitation data, as well as the limited set of variables available for multivariate analyses, our results indicate only weak long‐term tendencies toward an increase in the number of drier days per year, which should be interpreted as exploratory rather than predictive. Some studies analysing past climate using historical series or developing models to predict future conditions in the studied region report an increase in total rainfall (Oliveira et al. 2020; Sondermann et al. 2022), although our analyses focusing on the number of drier days per year suggest another aspect of the general climatic trends. By using the critical hydration thresholds for photosynthesis detected in this study, we identified a slight increase in the frequency of drier (precipitation < 2 mm) days. This finding suggests that even if total rainfall does increase in the region, it may become more temporally concentrated throughout the year, as mentioned by Sondermann et al. (2022). In this context, data related to variations in air humidity could be more sensitive in demonstrating local trends, even though their effects on cyanobacterial biofilms are likely to be indirect compared to precipitation data. The trend toward an increasing number of days per year with lower rainfall, lower relative humidity (below 60%), and higher VPD values particularly since the 1990s points to a shift in the region's moisture regime during the studied period. The quantity of water retained within the bark surface and associated biofilm after rainfall events was not quantified in the present study, but we generated relevant information about photosynthesis in the biofilms covering bark of *J. mimosifolia* under drier and wet conditions, suggesting changes in response to climate changes. Although cyanobacterial biofilms photosynthesize only in the presence of liquid water, relative humidity likely has an indirect effect by determining the duration of liquid water availability. To evaluate relevant changes in RH from 1961 to 2023, we need to establish a critical value for comparisons and our data suggested this as 60%, which was similar to limiting RH values (60%–70%) for cyanobacteria activity in terrestrial environments (Davila et al. 2013; Villa et al. 2015). This integration between the physiological and climatological data obtained in this study and the predicted climate scenarios in many regions indicates that the ecological performance of these communities and their ecological functions are likely to be negatively impacted where more frequent and prolonged dry periods are predicted. As the nitrogenase activity in aerophytic cyanobacteria is highly dependent on climate conditions (Qi et al. 2022), nitrogen fixation and primary production may decline, and secondary effects on the surrounding environments can be expected, although these relationships require more in‐depth investigation.

Although tolerant, our study demonstrates that photosynthetic functioning in bark biofilms was strongly associated with the hydration status of the bark‐associated matrix, with some indirect effects of RH. Rapid metabolic reactivation following water input supports the notion of pronounced physiological resilience, whereas reductions in environmental moisture were accompanied by a slower decline in photosynthetic activity.

## 5 Conclusion

Here, we defined the hydric conditions required to initiate the reestablishment of photosynthetic processes and quantified the temporal progression of recovery following hydration events. The dataset generated contributes to advancing the ecophysiological framework of terrestrial photosynthetic microorganisms and provides essential information for developing microbial conservation strategies. Future investigations should assess the combined influence of moisture dynamics with other stressors, including thermal variability and atmospheric pollutants, across different environmental contexts to strengthen ecological predictions and inform conservation policies under accelerating global climate change.

## Author Contributions

**Ana Beatriz de Lima Freitas:** investigation, writing – original draft, formal analysis, visualization, data curation. **Tiago Vilas‐Boas:** conceptualization, investigation, writing – review and editing, methodology, formal analysis, data curation. **Cleber Cunha Figueredo:** conceptualization, funding acquisition, writing – review and editing, visualization, methodology, project administration, supervision, data curation, resources.

## Funding

This work was supported by Brazilian Coordination for the Improvement of Higher Education Personnel (CAPES) and by the Research Support Foundation of the State of Minas Gerais (FAPEMIG).

## Conflicts of Interest

The authors declare no conflicts of interest.

## Data Availability

The data that support the findings of this study are available on request from the corresponding author. The data are not publicly available due to privacy or ethical restrictions.
